# Rare case of ruptured sinus of Valsalva aneurysm presenting only with diastolic murmur: cine mode reconstruction of cardiac computed tomography revealed flap motion of rupture site

**DOI:** 10.1093/ehjcr/ytz070

**Published:** 2019-05-18

**Authors:** Yu Yamada, Tomomi Koizumi

**Affiliations:** Department of Cardiovascular Medicine, National Hospital Organization Mito Medical Center, Sakuranosato, 280, Ibaraki-machi, Higashi-Ibarakigun, Ibaraki, Japan

A 62-year-old man with a history of ascending aortic replacement for aortic dissection 3 years ago was referred to our hospital with 3 months of dyspnoea. His past medical history of hypertension was well-controlled by medication. The patient is 173 cm in height and weighs 69 kg, and his physical examination showed holo-diastolic murmur (Levine III/VI) in the third left sternal border, neck vein distention, and leg oedema. His echocardiogram showed preserved left ventricular ejection fraction, right sided heart enlargement, and trivial aortic regurgitation, but no shunt flow was observed. Unfortunately, the patient refused transoesophageal echocardiography, the images of which would have been optimal to visualize and evaluate this patient’s cardiac structures. Thus, other tests were performed, including cardiac catheterization, showing step-up of blood oxygen in the right atrium, and a pulmonary-systemic flow ratio of 4.1. The left anterior oblique view of aortography showed a regurgitant jet from aorta to right atrium which occurred in diastole (Supplementary material online, *Video S1*), but the ruptured site remained still unclear. Therefore, electrocardiogram (ECG)-gated cardiac computed tomography (CCT) was done and revealed sinus of Valsalva aneurysm (SVA) of non-coronary cusp which ruptured to the right atrium (Figure *1*). Further, cine mode reconstruction of CCT showed flap motion of rupture site which opened only during diastole (*Figures 2* and *3*, Supplementary material online, *Video S2*). With an estimated aortic root diameter of 60 mm, the decision for the patient to undergo aortic root replacement was made. Following this surgery, the patient experienced a full recovery and was discharged from the hospital.

**Figure 1:**
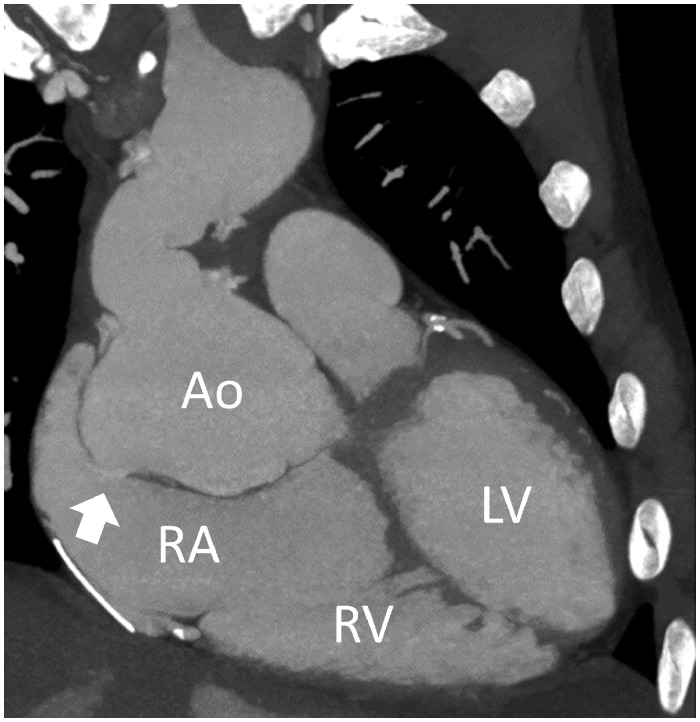
The coronal reconstruction of cardiac computed tomography showed dilated Valsalva sinus of non-coronary cusp which ruptured to the right atrium (white arrow). Ao, aorta; LV, left ventricle; RA, right atrium; RV, right ventricle.

**Figure 2:**
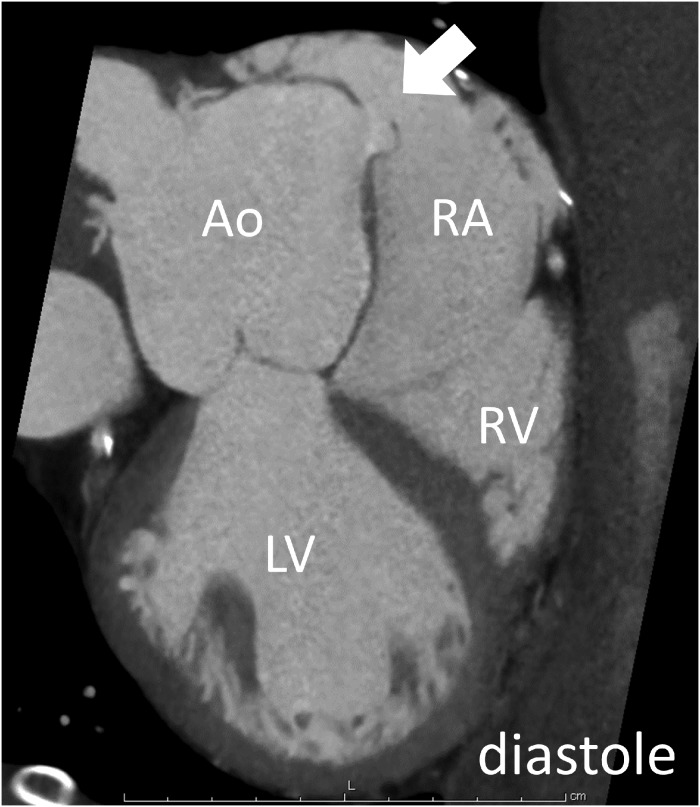
The cine mode reconstruction of cardiac computed tomography showed the flap-type rupture, which was opened to the right atrium in diastole. Ao, aorta; LV, left ventricle; RA, right atrium; RV, right ventricle.

**Figure 3:**
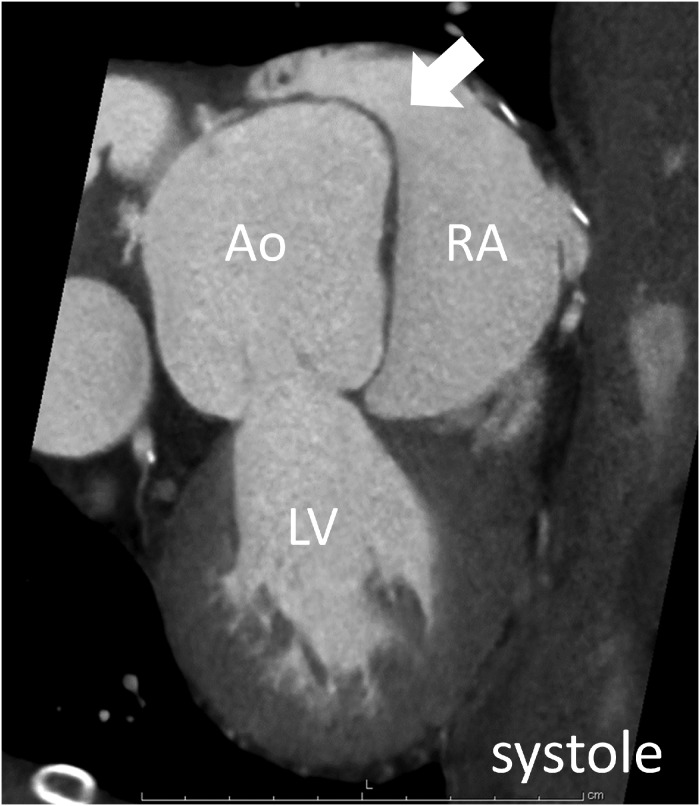
The cine mode reconstruction of cardiac computed tomography showed the flap-type rupture, which was closed in systole. Ao, aorta; LV, left ventricle; RA, right atrium; RV, right ventricle.

Sinus of Valsalva aneurysm is a congenital or acquired cardiac anomaly, yet the history of ascending aortic replacement might be considered as a risk for SVA in this case.^1^ Rupture of SVA usually occurs to the right atrium or ventricle, and typically causes a continuous murmur.^2^ Rupture of SVA with only a diastolic murmur, as shown in this case, is very rare. While ECG-gated CCT has already been established to diagnose SVA^3^, cine mode reconstruction of CCT was very useful for the detection of detailed flap motion at the rupture site which opened only during diastole. During systole, the flap might be closed because of low intra-Valsalva pressure (caused by Venturi effect created by the elevated cardiac output), whereas left-to-right shunt might have occurred only in diastole because the pressure in the aortic sinuses was maximal during diastole.

## Supplementary Material

ytz070_Supplementary_Video.zip
