# Supplementary material for: Rare case of ruptured sinus of Valsalva aneurysm presenting only with diastolic murmur: cine mode reconstruction of cardiac computed tomography revealed flap motion of rupture site
Source: Eur Heart J Case Rep. 2019 May 18;3(2):ytz070. doi: 10.1093/ehjcr/ytz070 (PMC6601151; doi:10.1093/ehjcr/ytz070)
Supplement: ytz070_Supplementary_Video.zip [file ytz070_supplementary_video.zip › ytz070-suppl_data/ytz070_Slide_Set.pptx]

## Slide 1
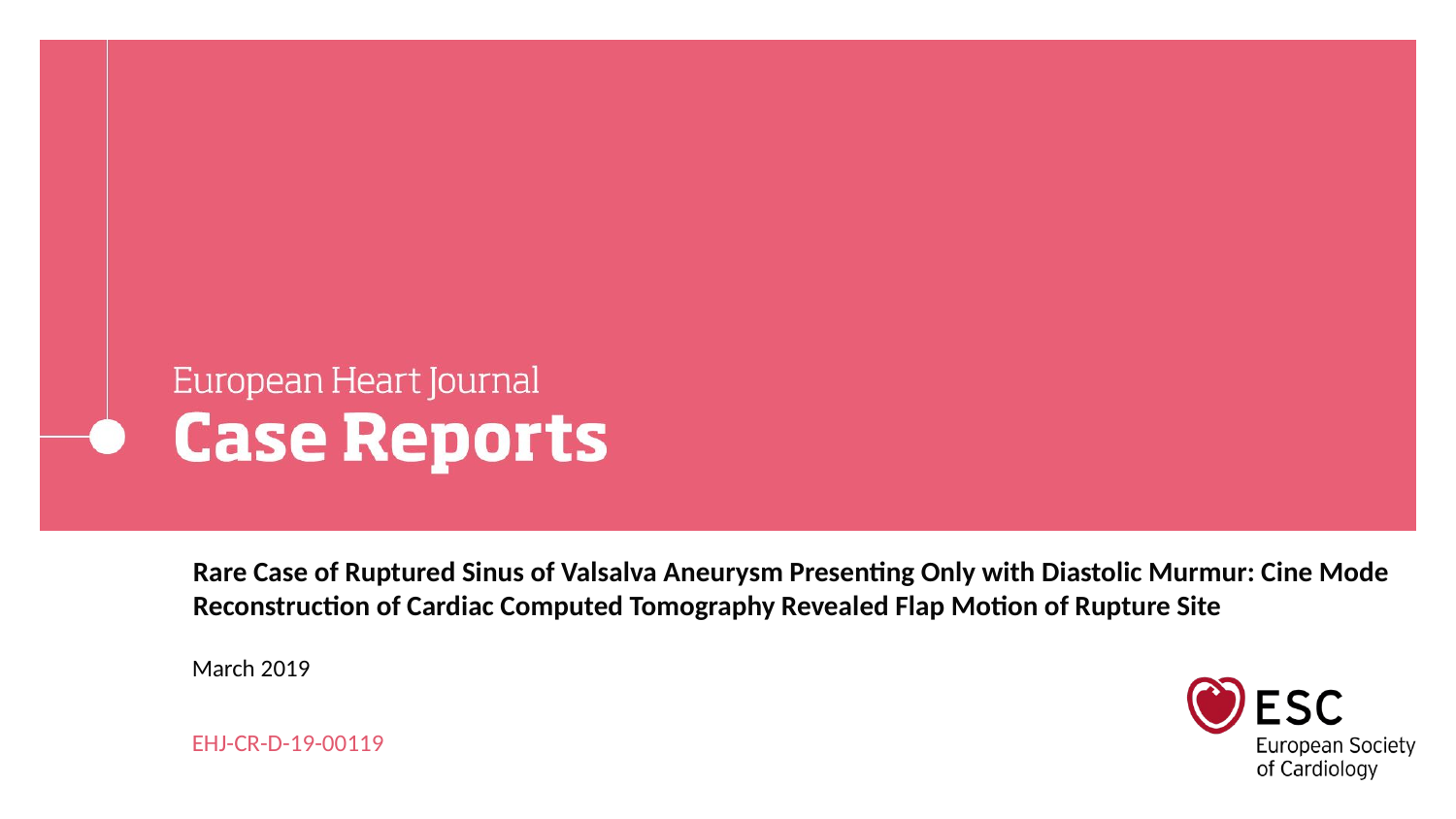

# Rare Case of Ruptured Sinus of Valsalva Aneurysm Presenting Only with Diastolic Murmur: Cine Mode Reconstruction of Cardiac Computed Tomography Revealed Flap Motion of Rupture Site
March 2019
EHJ-CR-D-19-00119

## Slide 2
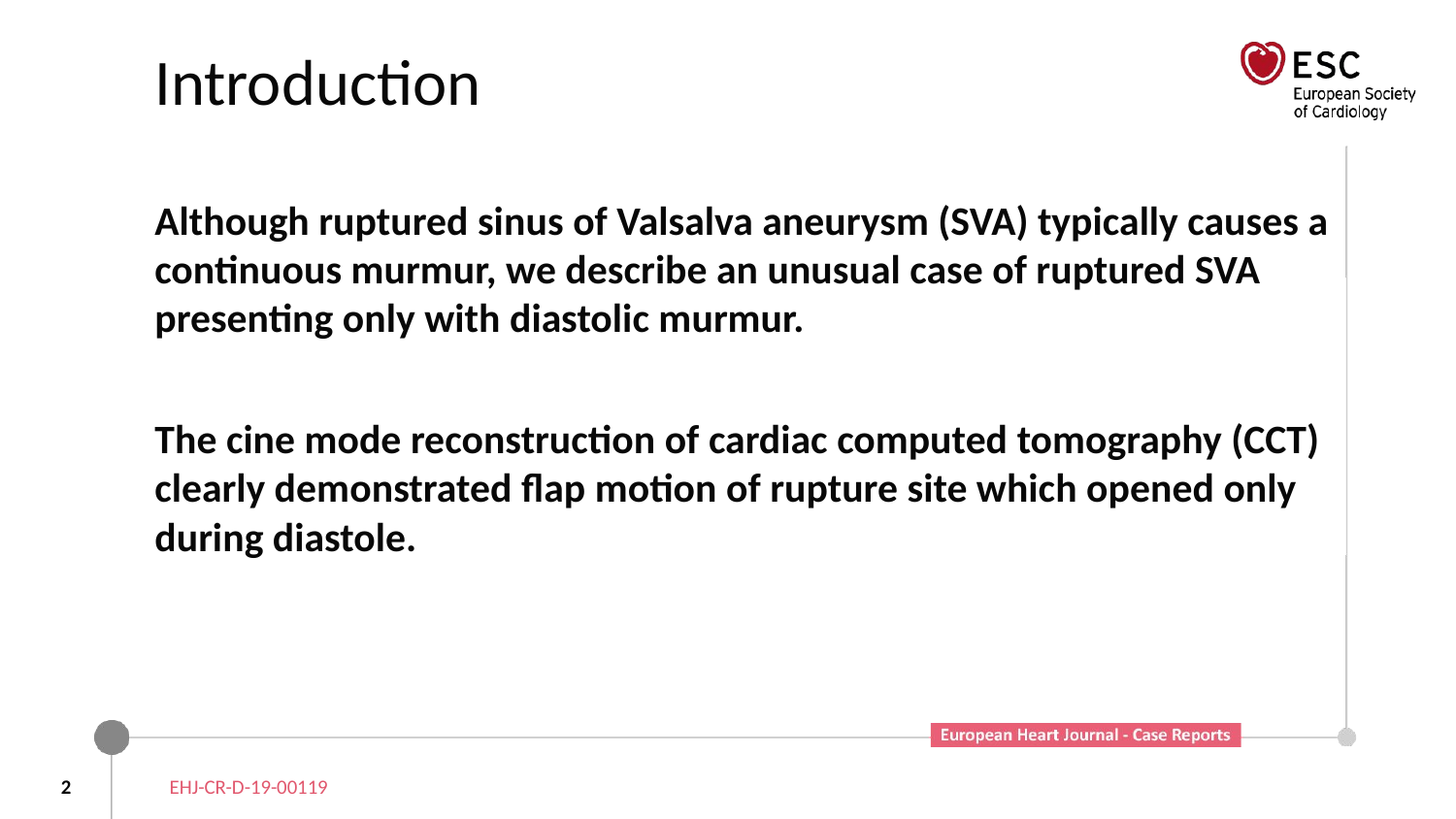

# Introduction
Although ruptured sinus of Valsalva aneurysm (SVA) typically causes a continuous murmur, we describe an unusual case of ruptured SVA presenting only with diastolic murmur.
The cine mode reconstruction of cardiac computed tomography (CCT) clearly demonstrated flap motion of rupture site which opened only during diastole.
2
EHJ-CR-D-19-00119

## Slide 3
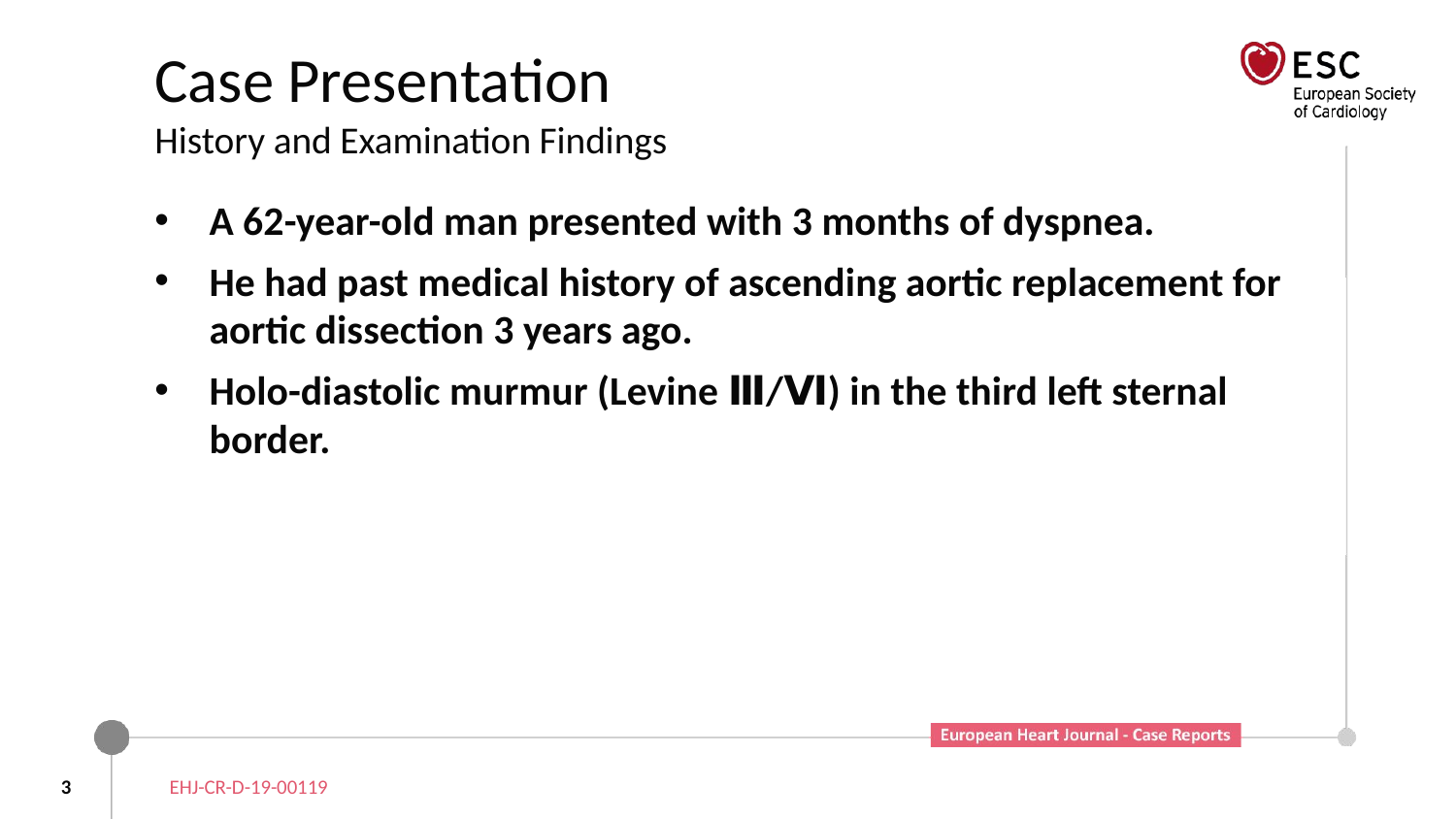

# Case Presentation History and Examination Findings
A 62-year-old man presented with 3 months of dyspnea.
He had past medical history of ascending aortic replacement for aortic dissection 3 years ago.
Holo-diastolic murmur (Levine Ⅲ/Ⅵ) in the third left sternal border.
3
EHJ-CR-D-19-00119

## Slide 4
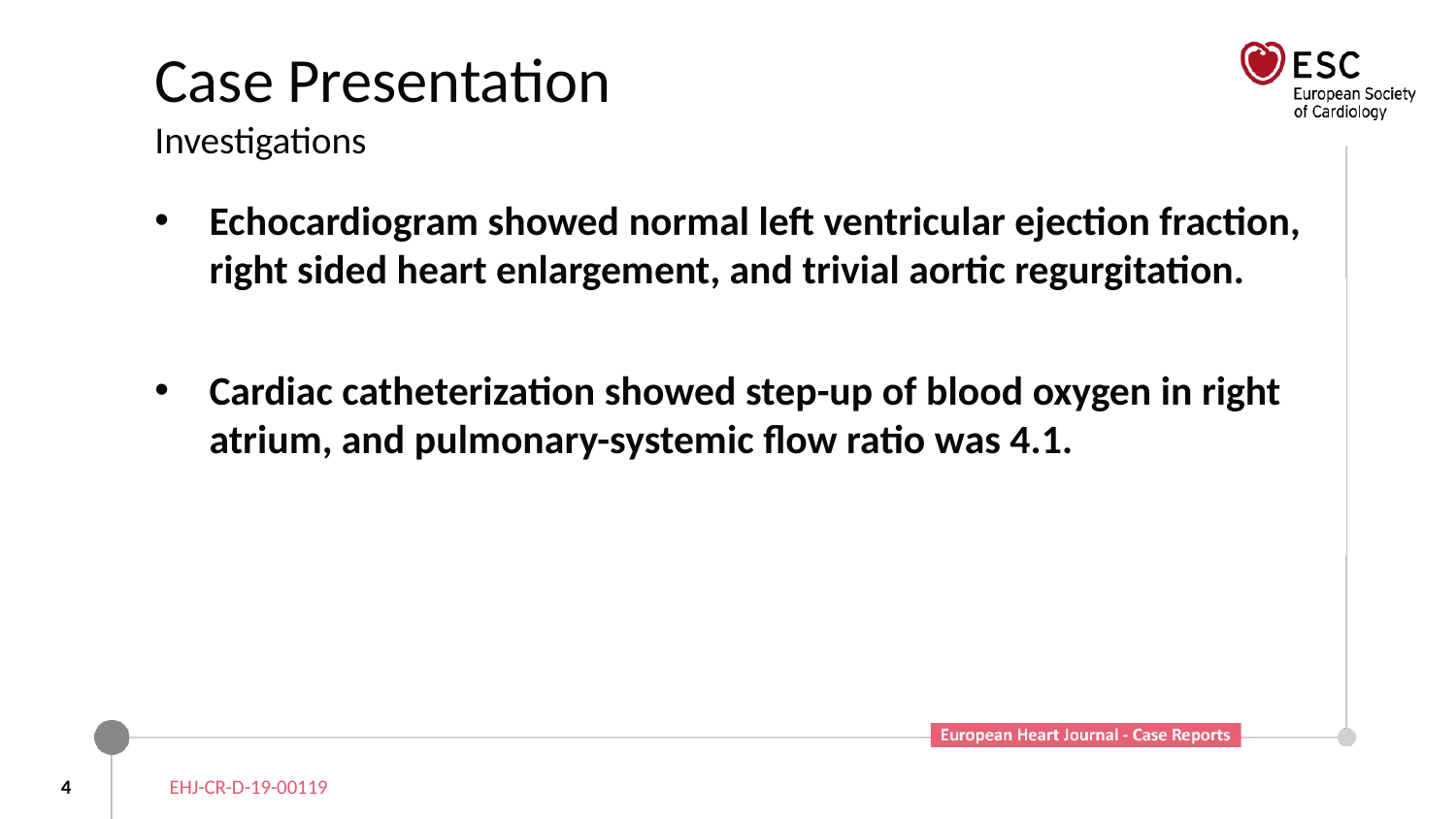

# Case Presentation Investigations
Echocardiogram showed normal left ventricular ejection fraction, right sided heart enlargement, and trivial aortic regurgitation.
Cardiac catheterization showed step-up of blood oxygen in right atrium, and pulmonary-systemic flow ratio was 4.1.
4
EHJ-CR-D-19-00119

## Slide 5
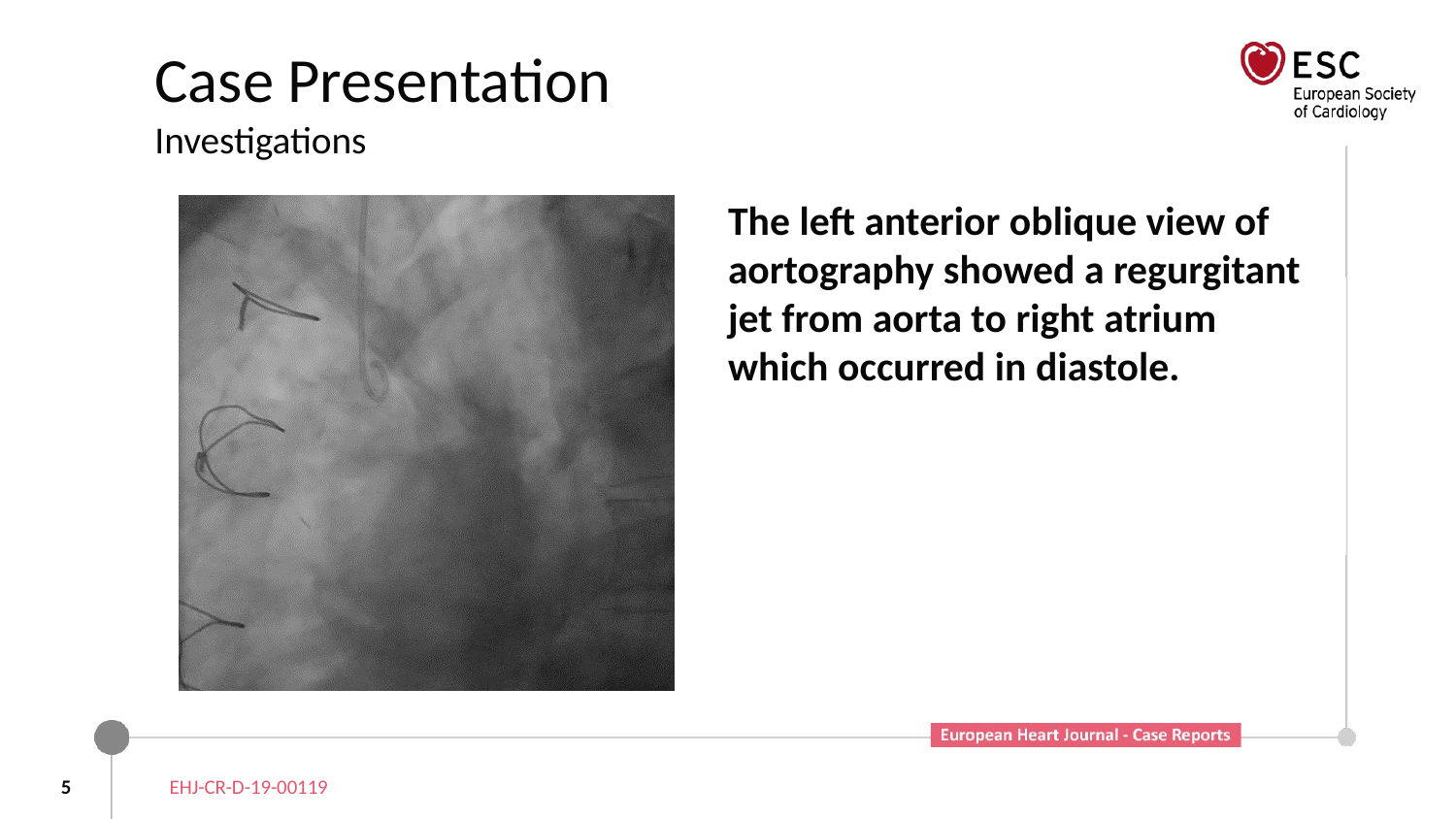

# Case Presentation Investigations
The left anterior oblique view of aortography showed a regurgitant jet from aorta to right atrium which occurred in diastole.
5
EHJ-CR-D-19-00119

## Slide 6
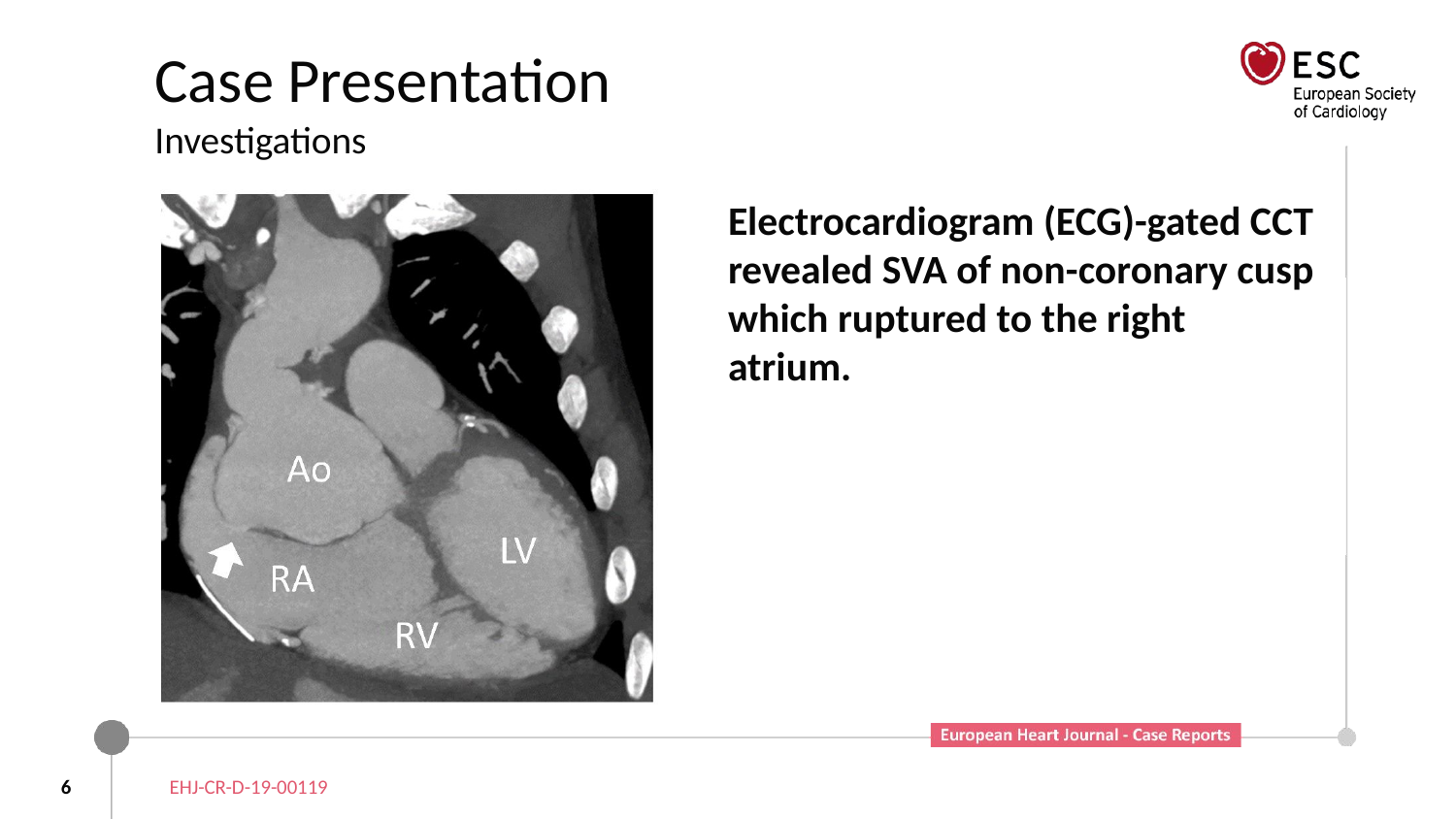

# Case Presentation Investigations
Electrocardiogram (ECG)-gated CCT revealed SVA of non-coronary cusp which ruptured to the right atrium.
6
EHJ-CR-D-19-00119

## Slide 7
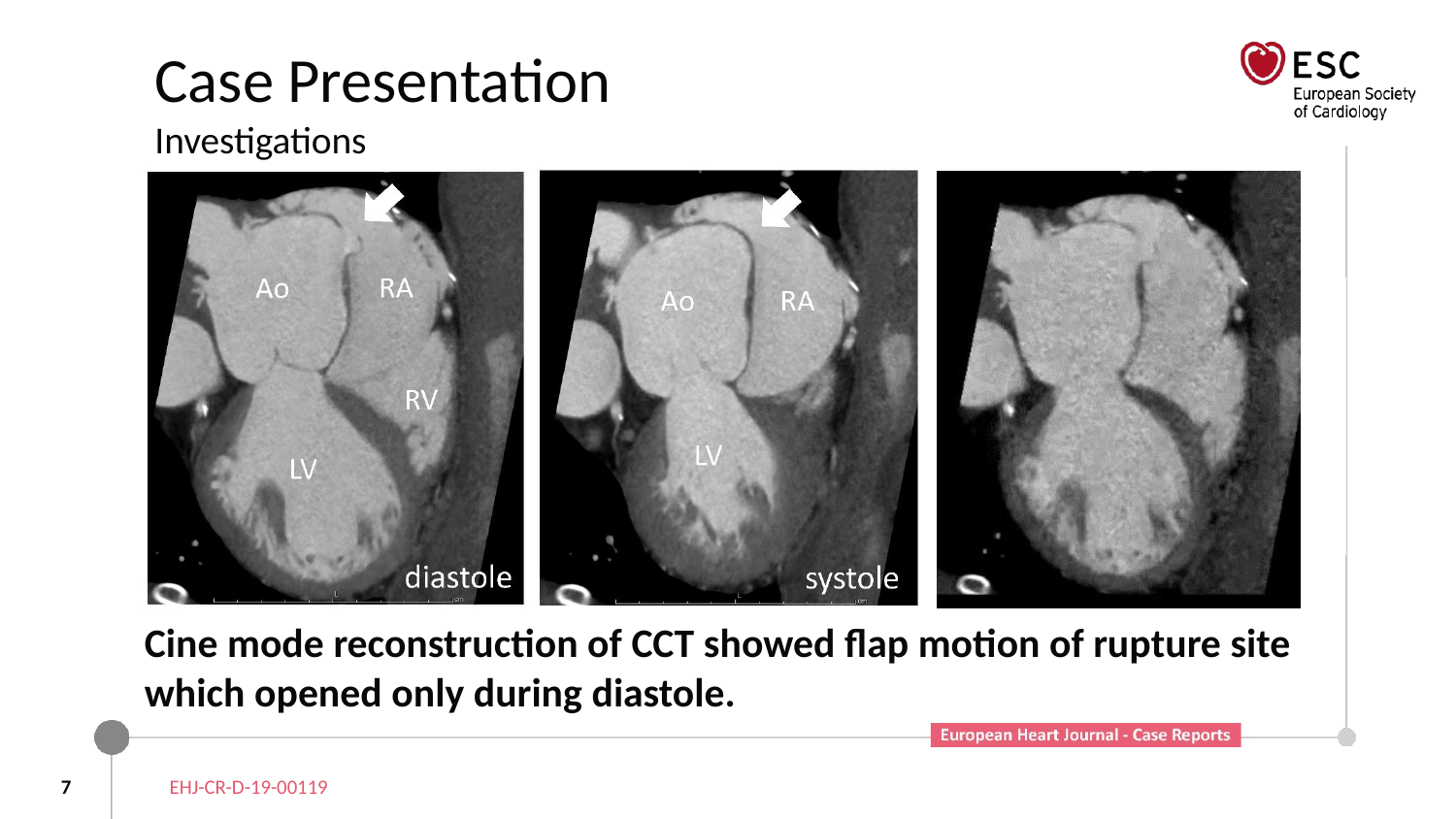

# Case Presentation Investigations
Cine mode reconstruction of CCT showed flap motion of rupture site which opened only during diastole.
7
EHJ-CR-D-19-00119

## Slide 8
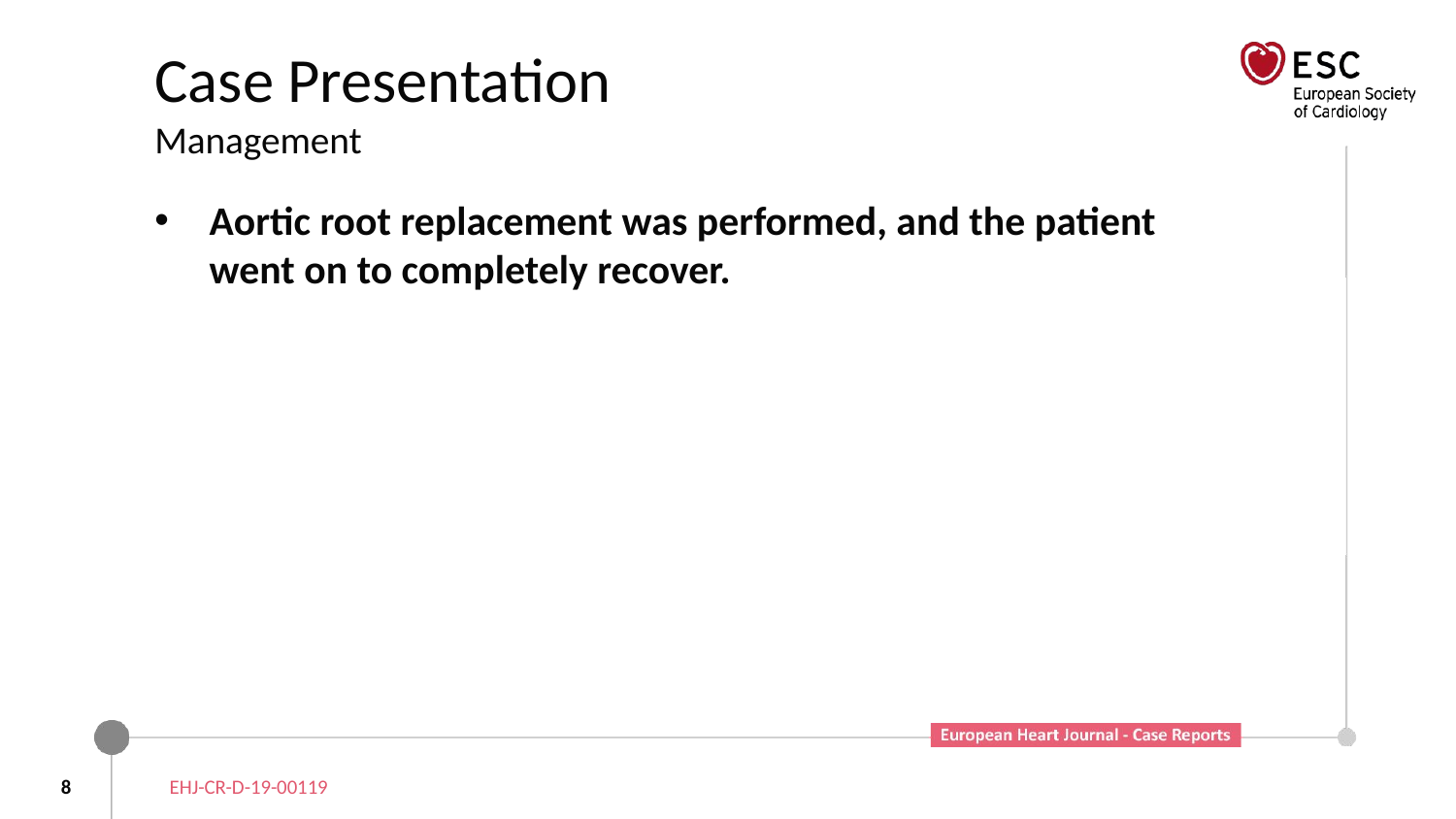

# Case Presentation Management
Aortic root replacement was performed, and the patient went on to completely recover.
8
EHJ-CR-D-19-00119

## Slide 9
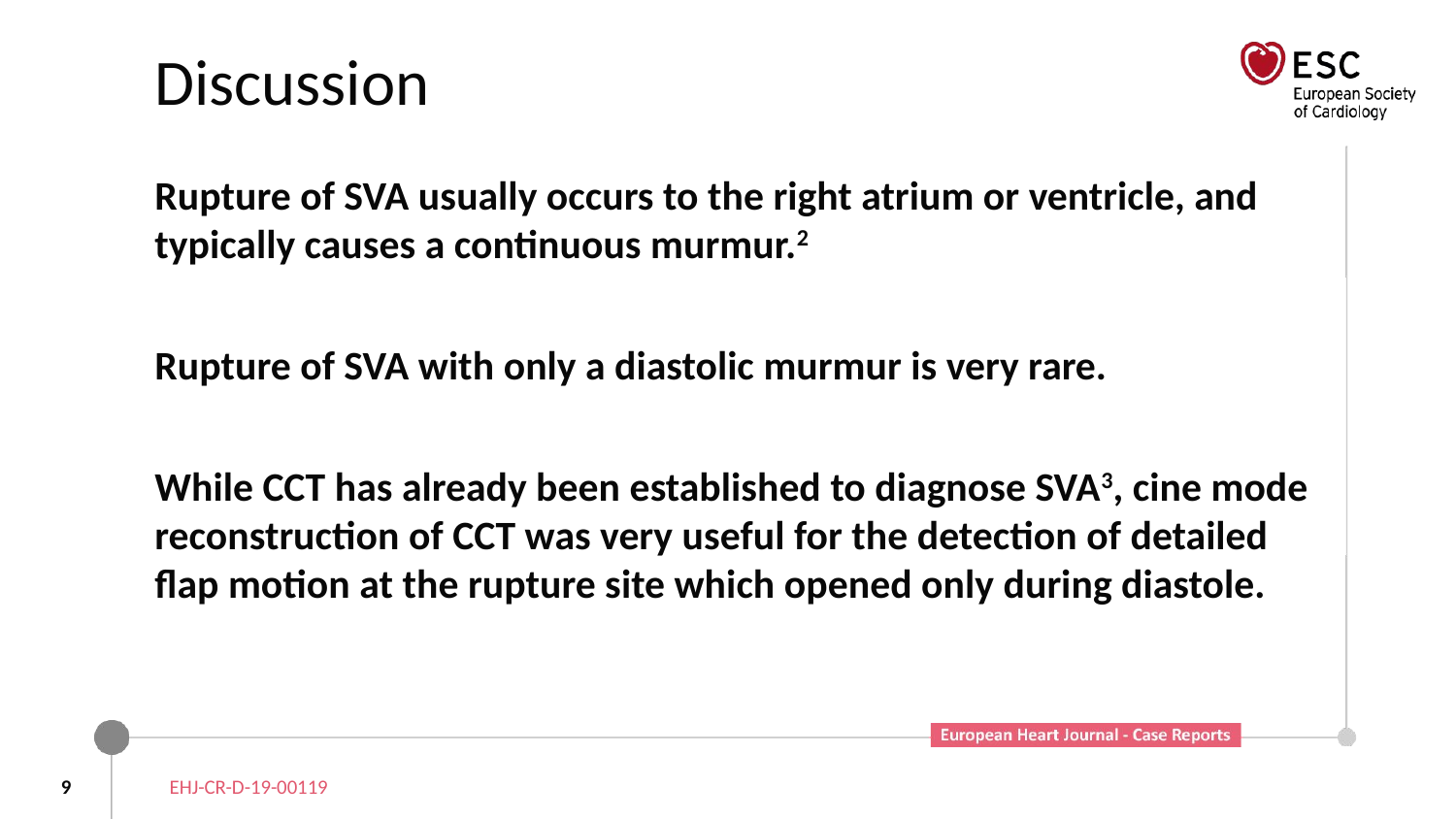

# Discussion
Rupture of SVA usually occurs to the right atrium or ventricle, and typically causes a continuous murmur.2
Rupture of SVA with only a diastolic murmur is very rare.
While CCT has already been established to diagnose SVA3, cine mode reconstruction of CCT was very useful for the detection of detailed flap motion at the rupture site which opened only during diastole.
9
EHJ-CR-D-19-00119

## Slide 10
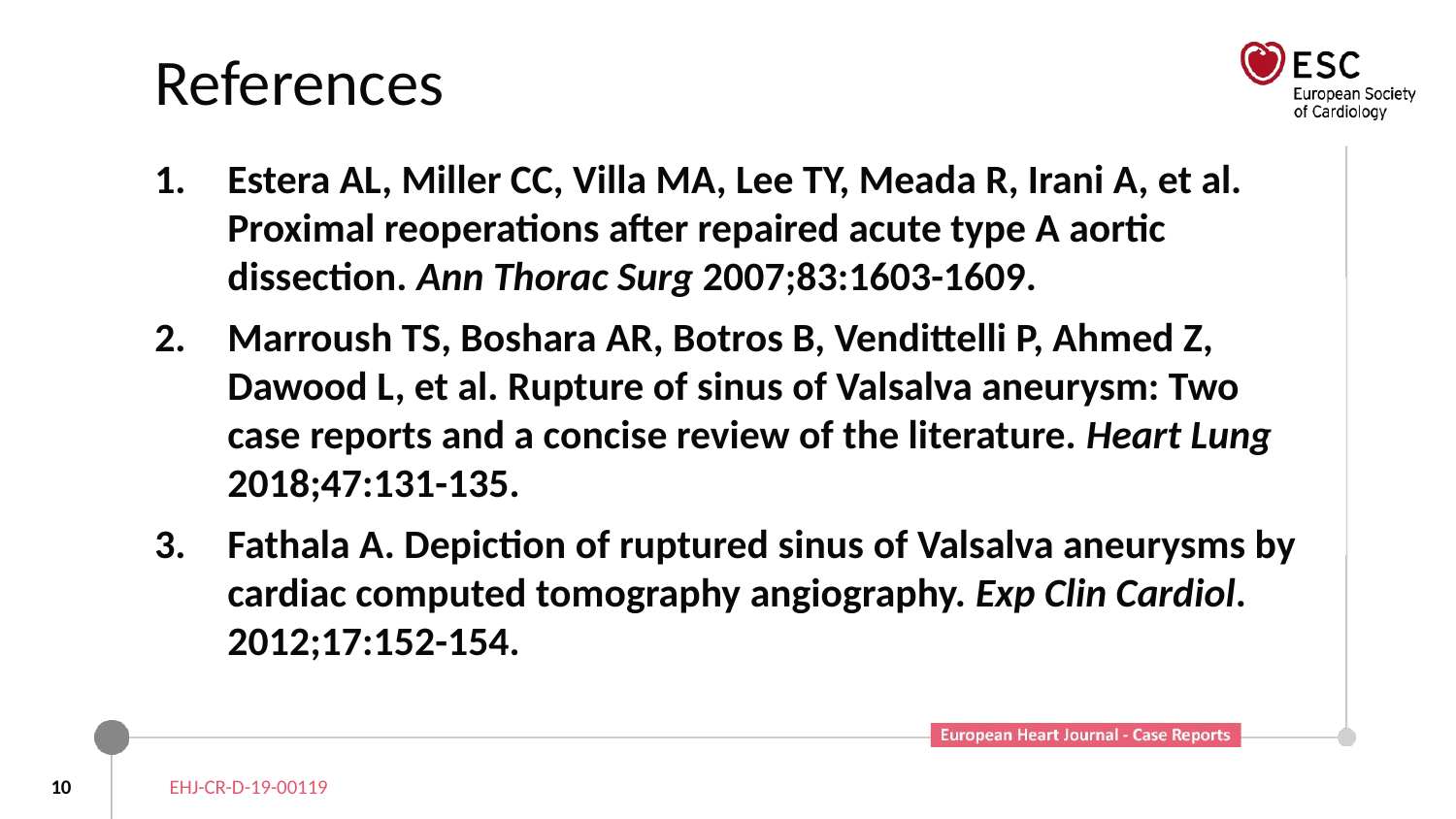

# References
Estera AL, Miller CC, Villa MA, Lee TY, Meada R, Irani A, et al. Proximal reoperations after repaired acute type A aortic dissection. Ann Thorac Surg 2007;83:1603-1609.
Marroush TS, Boshara AR, Botros B, Vendittelli P, Ahmed Z, Dawood L, et al. Rupture of sinus of Valsalva aneurysm: Two case reports and a concise review of the literature. Heart Lung 2018;47:131-135.
Fathala A. Depiction of ruptured sinus of Valsalva aneurysms by cardiac computed tomography angiography. Exp Clin Cardiol. 2012;17:152-154.
10
EHJ-CR-D-19-00119
